# Colorectal carcinomas in MUTYH-associated polyposis display histopathological similarities to microsatellite unstable carcinomas

**DOI:** 10.1186/1471-2407-9-184

**Published:** 2009-06-15

**Authors:** Maartje Nielsen, Noel FCC de Miranda, Marjo van Puijenbroek, Ekaterina S Jordanova, Anneke Middeldorp, Tom van Wezel, Ronald van Eijk, Carli MJ Tops, Hans FA Vasen, Frederik J Hes, Hans Morreau

**Affiliations:** 1 Center for Human and Clinical Genetics, Leiden University Medical Center, Leiden, The Netherlands; 2 Department of Pathology, Leiden University Medical Center, Leiden, The Netherlands; 3 Department of Gastroenterology, Leiden University Medical Center, Leiden, The Netherlands; 4 The Netherlands Foundation for the Detection of Hereditary Tumours, Leiden, The Netherlands

## Abstract

**Background:**

MUTYH-associated polyposis (MAP) is a recessively inherited disorder which predisposes biallelic carriers for a high risk of polyposis and colorectal carcinoma (CRC). Since about one third of the biallelic MAP patients in population based CRC series has no adenomas, this study aimed to identify specific clinicopathological characteristics of MAP CRCs and compare these with reported data on sporadic and Lynch CRCs.

**Methods:**

From 44 MAP patients who developed ≥ 1 CRCs, 42 of 58 tumours were analyzed histologically and 35 immunohistochemically for p53 and beta-catenin. Cell densities of CD3, CD8, CD57, and granzyme B positive lymphocytes were determined. *KRAS2*, the mutation cluster region (MCR) of *APC, p53*, and *SMAD4 *were analyzed for somatic mutations.

**Results:**

MAP CRCs frequently localized to the proximal colon (69%, 40/58), were mucinous in 21% (9/42), and had a conspicuous Crohn's like infiltrate reaction in 33% (13/40); all of these parameters occurred at a higher rate than reported for sporadic CRCs. Tumour infiltrating lymphocytes (TILs) were also highly prevalent in MAP CRCs. Somatic *APC *MCR mutations occurred in 14% (5/36) while 64% (23/36) had *KRAS2 *mutations (22/23 c.34G>T). G>T tranversions were found in *p53 *and *SMAD4*, although the relative frequency compared to other mutations was low.

**Conclusion:**

MAP CRCs show some similarities to micro-satellite unstable cancers, with a preferential proximal location, a high rate of mucinous histotype and increased presence of TILs. These features should direct the practicing pathologist towards a MAP aetiology of CRC as an alternative for a mismatch repair deficient cause. High frequent G>T transversions in *APC *and *KRAS2 *(mutated in early tumour development) but not in *P53 *and *SMAD4 *(implicated in tumour progression) might indicate a predominant MUTYH effect in *early *carcinogenesis.

## Background

MUTYH-associated polyposis (MAP) is an autosomal recessive disorder, which may be responsible for approximately 0.5–1% of colorectal carcinomas (CRCs).[1,2] Most biallelic *MUTYH *mutation carriers are reported to develop multiple polyps (typically between 10–500).[3,4] However, in seven population based CRC studies, 15 out of 39 (38%) proven biallelic *MUTYH *mutation carriers had no polyps besides their CRC while seven (18%) had a limited number of adenomas (i.e. <10). [1,2,5-9] Therefore, the practicing pathologist should also consider biallelic *MUTYH *mutations in CRC patients with none or less than 10 polyps.

The MUTYH protein is a base excision repair (BER) glycosylase involved in the repair of DNA damage resulting from the oxidation of guanine nucleotides. The oxidation product of guanine, 8-oxo-7,8-dihydro-2'-deoxyguanosine (8-oxoG), readily mispairs with adenosine nucleotides during DNA replication. MUTYH acts by scanning the newly synthesized DNA strand for any mispaired adenines, either with guanines or 8-oxoG's, and excising them. A dysfunctional MUTYH protein increases the occurrence of somatic G>T transversions. For instance, somatic mutations in the *APC *gene in MAP tumours involve almost exclusively G>T transversions, an observation that led to the discovery of the MAP syndrome.[10] Similarly, the most prevalent *KRAS2 *mutation in MAP tumours is a G>T transversion at codon 12 (c.34G>T), which was reported to be present in 64% of MAP carcinomas.[11] Such mutation is infrequent in sporadic CRCs, according to published consecutive series.[12]

Recently it was reported that MAP CRCs are often near-diploid (52%) and commonly contain chromosomal regions of copy neutral loss of heterozygosity (LOH) (71%). In copy-neutral LOH there is no loss of genetic material and this can arise via mitotic recombination, non-disjunction, or deletion and reduplication events. This is in contrast to sporadic colon cancer, where physical loss of genetic material is the main characteristic.[13]

Another set of CRCs with deficiencies in DNA repair are the mismatch repair deficient tumours. Mismatch repair deficient CRCs with high-microsatellite instability (MSI-high) arise in the context of the Lynch syndrome or have a sporadic origin due to somatic inactivation of *hMLH1*. MSI-high tumours have characteristic histological and molecular features: they are most often near-diploid and arise preferentially in the right side of the colon. They have a high prevalence of mucinous and medullary histotypes, poor differentiation, a Crohn's-like lymphocytic reaction, and a high amount of (intra-epithelial) tumour infiltrating lymphocytes (TILs). These characteristics are currently employed for diagnostic purposes and may have implications for patient treatment and prognosis. [14-17]

In the present study we describe histological and molecular aspects of MAP carcinomas in a Dutch cohort, and compare these with data available in literature of consecutive series of sporadic, MSI-high (sporadic), and Lynch syndrome-derived CRCs. The aim of this study was to identify specific characteristics that would aid in the diagnosis of MAP CRCs and in differentiation from carcinomas arising from distinct genetic backgrounds.

## Methods

### Patients

Fifty-seven MAP families, from which clinical data were available, were studied. Informed consent was obtained according to protocols approved by the Leiden University Medical Center ethics review board (02-2004). These families include 57 index-patients with biallelic homozygous or compound heterozygous *MUTYH *mutations and 22 siblings with biallelic *MUTYH *mutations. Forty index-patients have been described previously in lesser detail.[3] One patient was reported to have CRC stage A according to the modified Astler-Coller guidelines at age 21, but after re-evaluation appeared to have high grade dysplasia (carcinoma *in situ) *and was not included in this series of MAP carcinomas. To date, 56% (44/79) of the carriers have developed CRC. A total of 58 CRCs were diagnosed in these 44 MAP patients, composed of 26 males and 18 females. Hematoxylin and eosin (H&E) stained slides and tissue material from 42 and 38 CRCs, respectively, belonging to 35 MAP patients, could be retrieved from 23 pathology laboratories throughout The Netherlands.

### Histological examination

Histological tumour type and grade were independently assessed by two observers (HM and MN) according to the World Health Organization classification.[18] Staging was performed according to the modified Astler-Coller (MAC) and the American Joint Committee on Cancer (AJCC) TNM staging system guidelines. Metachronous tumours were defined as new tumours arising in the colon at least six months after the initial diagnosis.[19] Semi-quantitative assessment defined two subgroups, according to mucinous content of the tumour: >50% of tumour area involved (mucinous) and 10 to 50% of tumour area involved. Mucinous adenocarcinomas and signet-ring cell carcinoma by convention were considered poorly differentiated [18], although are also separately scored. A Crohn's-like reaction was assessed as grade 0, 1+, or 2+.[20] Grade 2+ was referred to as conspicuous. Leukocyte infiltration was assessed semi-quantitatively on H&E-slides as none, moderate (visible only on high magnification (×40), or marked (visible already on low magnification ×10).

### Molecular analysis

Genomic DNA of paired normal colon and colorectal carcinoma tissue was isolated from formalin-fixed paraffin-embedded material, as described previously.[21] The percentage of tumour cells in the areas from which the punches were taken, were in all cases above 50% and in most cases above 70%. Microsatellite instability analysis was done according to the Bethesda guidelines using the markers D2S123, D5S346, D17S250, BAT25, BAT26, and BAT40. A tumour was scored MS-stable when no marker showed instability, MSI-high when > 30% of markers showed instability, and MSI-low when only one marker (<30%) showed instability.[22] For somatic mutation analysis of *APC, KRAS2, p53 *and *SMAD4 *DNA sequence analysis was performed. Codon 12 and 13 of the *KRAS*2 gene and the *Mutation Cluster Region *(codons 1286–1513) of the *APC *gene were analyzed as described previously. [23] Primer pairs were designed for the coding regions and exon-intron boundaries of *p53 *exons 5–8 and *SMAD4 *exons 3–13. Primer details are available from the authors upon request. Germline DNA mutation analysis of the whole *MUTYH *gene was performed on lymphocytic DNA or DNA from formalin-fixed paraffin-embedded normal tissue as described previously.[3,5] Primer details are available from the authors upon request. For further details see the website of our DNA diagnostic laboratory [24]. To describe *MUTYH *mutations we used the most up-to-date annotation, see the LOVD database.[25,26]

### Immunohistochemical analysis using tissue microarray

To construct a tissue microarray (TMA), triplicate tissue cores were taken from tumour tissue as described previously [27]on the basis of H&E-stained slides reviewed by a pathologist (HM).) Sections were deparaffinised and endogenous peroxidases were inactivated with 0.3% H_2_O_2 _in methanol solution after antigen retrieval by means of microwave oven treatment for 10 minutes in 10 mM citrate buffer pH 6.0 (p53, MLH1) or 1 mM Tris-EDTA pH 8.0 (beta-catenin, PMS2). Sections were incubated overnight at room temperature with mouse anti-human monoclonal antibodies directed against p53 (clone D0–7, 1:1000, Neomarkers, USA), MLH1 (clone G168–728, 1:50, BD Pharmigen, USA), PMS2 (clone A16-4, 1:200, BD Pharmigen) and beta-catenin (encoded by *CTNNB1) *(clone 14, 1: 800, BD Transduction Laboratories, USA). The following day, tissue sections were incubated with a biotinylated secondary antibody in PBS/BSA 1%. Diaminobenzidine tetrahydrochloride was used as a chromogen for the development of the staining. The slides were counterstained with hematoxylin. Immunohistochemistry (IHC) was scored for p53 nuclear staining as: 1 = none, 2 = >0<25% (mostly indicative of a functional intact p53 status), 3 = 25%–75%, or 4 = >75% (the latter two indicative of p53 dysfunction). Staining of beta-catenin was graded by the following scale: 1 = membranous staining, 2A = membranous and some nuclear staining, 2B = membranous staining, and increased nuclear staining, 3 = strong nuclear staining, with less or no membranous staining. Normal epithelium and stromal cells provided positive internal controls.

### Infiltrate analysis using fluorescent immunohistochemical staining

Fluorescent immunostaining was performed as previously described.[28] Five μm TMA sections were used in all experiments. A mixture of the antibodies ab828 (rabbit polyclonal, anti-CD3; Abcam, UK), hNK-1 (mouse monoclonal IgM, anti-CD57; Department of Pathology, LUMC, The Netherlands), and 4B11 (mouse monoclonal IgG2b, anti-CD8; Novo Castra, UK) was added to each slide. The next day slides were incubated with the appropriate combination of fluorescent antibody conjugates (IgG-Alexa Fluor 546, IgM-Alexa Fluor 488 and IgG2b-Alexa Fluor 647). Alexa Fluor conjugates were obtained from Molecular Probes (Leiden, the Netherlands). The images were captured with a confocal laser scanning microscope (LSM) (Zeiss LSM510, Zeiss, Germany). The number of each leukocyte sub-type was assessed per tumour area (TILs/mm^2 ^tumour epithelium) using the LSM software (Zeiss). Cells staining for CD3, and not for CD8 or other CD markers, were considered to be T-helper lymphocytes. Standard immunostaining for the associated cytotoxic molecule granzyme B (NCL-GRAN-B; clone 11F1; Novo Castra) was performed in sequential sections. To calculate the density of granzyme B positive cells we used the assessed tumour area from the fluorescent immunostaining.

### Literature on histological and molecular features of CRC

We compared literature available, describing histological and molecular features, on sporadic, MSI-high and Lynch CRCs. The following terms were employed as search terms: colon carcinomas, bowel cancer, CRC, sporadic, MSI-high, Lynch, HNPCC, histological, molecular, APC, KRAS2, p53, beta-catenin, CTNNB1, SMAD4, tumour infiltrating lymphocytes, tumour infiltrating lymphocytes and intra-epithelial lymphocytes. All relevant references within articles were identified and included.

### Statistical Analysis

Fisher exact test was used to estimate an association between molecular and clinical-pathological parameters. The Spearman test was used to assess correlations. All *P*-values are reported for a two-tailed test; *P*-values of less than 0.05 were considered to be statistically significant. A group with high and low intraepithelial leukocyte infiltration was distinguished, using the median value as cut-point of the leukocyte-infiltration scores of all patients. When a patient had more than one carcinoma the mean number of lymphocytes in these carcinomas was used for the survival analysis. The Kaplan-Meier method was used to calculate the overall survival and the log-rank test was used for comparison of the survival curves. All statistical analyses were carried out using the SPSS software package (SPSS Inc. 12.0, USA).

## Results

### Histopathology of MAP carcinomas

**Figure 1:**
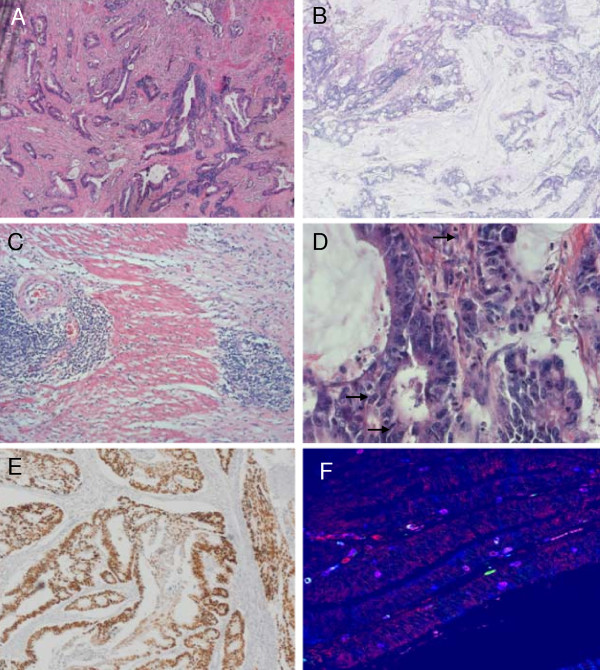
**MAP CRC histology**. A) moderate differentiation, tumour 23, 5× B) >50% mucinous, tumour 29, 5× C) Crohn's like infiltrate, tumour 34, 5× D) TILs, tumour 33, 40× E) p53 staining, >75% nuclear staining, tumour 32, 5× F) CD3/CD8/CD57 immunofluorescent staining, tumour 35, red cells: CD3^+^, purple cells: CD3^+^CD8^+^, white cells: CD3^+ ^CD8^+ ^CD57^+^, 40×.

Forty-four MAP patients were diagnosed with 58 CRCs (see Table 1) at a mean age of 49 years (Table 2 and 3). Ten patients (23%) had metachronous or synchronous carcinomas. The majority of carcinomas (35/51, 69%, metachronous tumours not included), were right-sided, i.e. proximal to the flexura lienalis. The majority of proximal tumours were located in ascending colon or cecum (80%, 28/35) and only a minority occurred in the transverse colon or hepatic flexure (14%, 5/35). Fifty-five percent (29/53) were stage B according to MAC guidelines or T>1N0M0 according to the TNM guidelines. Forty-two colon carcinomas from 35 MAP index patients were available for further study (Table 1). Histological analysis showed poor differentiation in 26% (11/42) and moderate in 71% (30/42, Figure 1A), mucinous CRCs represented 81% of these cases (9/11, Figure 1B). No MAP carcinomas showed a solid or medullary histotype or contained signet ring cells. A Crohn's like infiltrate was present in 33% (13/40, Figure 1C). In 40% (16/40) of tumours, focal necrosis (dirty necrosis within glandular lumina) was present. Tumour infiltrating lymphocytes assessed on H&E-slides were present in 74% (31/42) and were marked in 17% (7/42, Figure 1D). In 33 tumours MSI status was analyzed and all carcinomas but one were MS-stable. The latter carcinoma showed instability of less than 30% of markers (one dinucleotide marker) designating the tumour as MSI-low. Furthermore, staining for MLH1 and PMS2 was positive for all the tumours.

**Table 1:** MAP patients and histological features of colorectal carcinomas

*TumourNr*	*Fam* *nr*	*MUTYH* *Mut 1*^*a*^	*MUTYH* *Mut 2*	*Loc CRC*	*Age CRC*	*TNM stage*	*MAC stage†*	*Differentiation†† *	*Mucinous*	Chrohn's like infiltrate*	TILs**	*Nec-* *rosis~ *	Remarks/other cancers
1	61883.1	p.R247X	p.P405L	C	48	T4N0M0	B3	mod	No	1	no	yes	

2	19036.2	p.Y179C	p.Y179C	C	41	T2N2M1	D	mod	No	0	no	no	

3				C	41	T2N2M1	D	mod	No	2	no	no	

4				R/S	41	T2N2M1	D	muc	yes, >50%	1	no	no	

5				AC	41	T2N2M1	D	muc	yes, >50%	1	no	yes	

6	19049.1	p.Y179C^b^	p.Y179C	SF	56	T3N0M0	B2						Laryngeal carc. age 57

7				R	69	T3N0M0	B2	mod	No	2	+	no	Urothelial carc. age 67

8	19049.2	p.Y179C	p.Y179C	AC	53	T3N0M0	B2	mod	yes, <50%	2	++	no	

9	19053.4	p.Y179C^b^	c.933+3A>C	AC	51	T3N0M0	B2	mod	No	0	+	yes	

10	19053.2	p.Y179C^b^	c.933+3A>C	C	53	T3N0M0	B2	mod	No	2	+	yes	

11	19221.1	p.Y179C	p.P405L	C	56	T3N0M0	B2	muc	yes, >50%	0	+	yes	Duodenal carc age 56

12	20090.1	p.Y179C	p.P405L	AC	50	T3N0M0	B2	mod	yes, <50%	2	++	yes	Exo1 mutation IVS12-1 G>C (Jagmohan et al.)

13	20090.6	p.Y179C	p.P405L	R/S	39	T3N0M1	D	poor	No	0	no	yes	Exo1 mut. IVS12-1G>C

14	52240.1	p.P405L	p.P405L	R	58	T3N0M0	B2	muc	yes, >50%	0	+	no	Breast carc age 54

15	52596.1	p.Y179C	p.Y179C	C	44	T3N0M0	B	muc	yes, >50%	2	++	yes	

16	52654.1	p.P405L	p.P405L	AC	37	T1N2M0	C1	mod	No	2	+	no	

17				AC	37	T2N2M0	C1	mod	No		+	no	

18	53029.1	p.Y179C^b^	p.P405L	C	41	T3N2M0	C2	mod	No	1	+	no	

19	53231.1	p.G396D	p.G396D	AC	59	T2N0M0	B1	mod	No	0	+	yes	

20	54092.2	p.G396D^b^	p.G396D	C	60	T2N0M0	B1	mod	No	1	+	no	

21	54178.1	p.G396D	p.P405L	S	44	T3N0M0	B2	muc	yes, >50%	2	no	no	

22	54186.1	p.Y179C	p.Y179C	AC	45	T4NxM1	D	mod	No	0	+	no	Basal cell carc. age 42

23	54186.6	p.Y179C^b^	p.Y179C	S	43	T3N0M0	B2	mod	No	0	no	no	

24				HF	43	T3N0M0	B2	mod	yes, <50%	1	+	no	

25	54245.1	p.Y179C	p.Y179C	R	54								Breast carc age 77&79

26				'left'	57								Basal cell carc. age 53

27				I	77								

28				AC	77	T3N0M0	B2	mod	yes, <50%	1	+	no	

29	54962.10	p.G396D^b^	p.P405L	AC	51	T2N1M0	C1	muc	yes, >50%	0	++	no	

30	55247.1	p.Y179C	p.R247X	C	46	T2N0M0	B1	muc	yes, >50%	2	++	yes	

31	55535.1	p.Y179C	p.Y179C	C	45	T3N2M1	D	mod	yes, <50%	2	+	yes	

32	56081.1	p.Y179C	p.G396D	HF	59	T3N0M0	B2	mod	No	2	no	yes	

33	56081.2	p.Y179C^b^	p.G396D	AC	49	T3N1M0	C2	mod	yes, <50%	2	++	yes	

34	56566.1	p.Y179C	p.G396D	TC	67	T3N1M0	C2	mod	No	1	+	yes	

35	56641.1	p.Y179C	p.G396D	C	43	T3N0M0	B2	mod	yes, <50%	2	+	no	

36				TC	46	T3N0M0	B2	mod	No	1	+	no	

37	57139.1	c.1145delc	p.G396D	S	42	T2N0M0	B	mod	No	1	no	no	

38				S	42	T1N0M0	A	mod	yes, <50%		+	no	

39	57246.1	p.Y179C	p.Y179C	C	65	T2N0M0	B1	mod	yes, <50%	0	+	no	Duodenal carc age 64

40				AC	65	T2N0M0	B1	well	No	0	++	no	

41	57249.1	p.Y179C	p.Y179C	R/S	49	T3N0M0	B2	mod-poor	No	1	+		Esophageal carc. (Barret) age 59

42	57249.13	p.Y179C^b^	p.Y179C	R	52	T2N0M0	B1	mod	No	0	+		

43	57249.9	p.Y179C	p.Y179C	C	49	T2N1M0	C1	mod	No	1	no	no	

44	60322.4	p.Y179C	p.G396D	'right'	39	T4N1M0	C3	mod	No	0	+	yes	

45	52638.4	p.G396D^b^	p.R109W	C	52	T3N0M1	D	poor	No	1	+	yes	

46	57449.1	p.Y179C	p.Y179C	HF	45	T3N0M0	B2	muc	yes, >50%	0	+	no	2^nd ^carc.(rectum) age 69

47	19047.1	p.G396D	p.G396D	'right'	70	TxN1M0	C						

48	19106.1	p.Y179C	p.Y179C	R	40	TxN0M0	B						

49	57591.1	p.P405L	p.Y179C	C	40	TxN1M1	D						

50	60406.1	p.E480del	p.E480del	AC	51	T3N0M0	B2						

51	51063.1	p.Y179C	p.Y179C	SF	44	T1N0M0	A						

52	54140.1	p.E410fs	p.E410fs	R	42	TxN0M0	B						Cervical carc. age 27

53	57135.1	p.Y179C	p.Y179C	HF	46	TxN1M1	D						

54	55356.1	c.1145delc	p.P405L	R	42	TxN0M0	B						

55	53276.1	p.G396D	p.P405L	C	48	T1N0M0	A						Basal cell carc. age 58

56	19247.3	p.Y179C^b^	p.Y179C	R	43								CRC metastasis age 63

57				'right'	53	TxN1M1	D						

58				'right'	59								

Blank cells: not done/not ascertainable, CRC = colorectal cancer, R = rectum, S = sigmoid colon, DC = descending colon, SF = splenic flexure, TC = transverse colon, HF = hepatic flexure, AC = ascending colon, C = cecum, I = ileum.

^a^According to the recently changed *MUTYH *Nomenclature (Human Genome Variation Society), adding 14 amino acids after amino acid position 53. For example: Y165C>Y179C and G382D>G396D, ^b^analysis in DNA from FFPE material. **†**MAC = Modified Astler-Coller, **††**mod = moderate, muc = mucinous, *0 = none, 1 = mild reaction, 2 = intense reaction (conspicuous),** tumour infiltrating lymfocytes 0 = none/few, + = moderate, ++ = marked, ~yes = focal necrosis (dirty necrosis within glandular lumina).

**Table 2:** Histological and molecular features of carcinomas

Characteristics Colon carcinomas		MAP	Sporadic CRC	Sporadic MSI-high	Lynch (based on MMR mutations)
**Average age at CRC**		49 years [*CS*] 59 years^11^	68 years	67–75 years	47 years

**TNM stage**	**III or IV**	34% (51/148) 55% (10/18)[1]	42% (1781/4193)	43% (19/44)	15% (15/101)

**Proximal location**		69% (35/51) [*CS*] 29% (7/24)^11^ 43% (16/37)^4^ 46% (6/13)^31^	23% (1887/8129)	75% (308/411)	67% (74/111)

**(Meta) synchronous CRC**		23% (10/44) [*CS*] 33% (6/18)^11^ 26% (8/29)^4^	2% (14/832)		18% (7/38)

**Poor Differentiation**		26% (11/42) [*CS*] 22% (5/23)^11^ 100% (16/16)^31^	10% (765/7590)	41% (203/501)	38% (38/101)

**Mucinous**	**(>50%)**	21% (9/42) [*CS*] 13% (3/23)^11^ 0% (0/16)^31^	12% (292/2480)	28% (104/376)	35% (40/113)

**Crohn's like** **infiltrate**	**Conspicuous**	33% (13/40) [*CS*] 31% (5/16)^31^	28% (586/2059)	54% (318/589)	49% (37/76)

**Necrosis**		40% (16/40) [*CS*]	77% (356/465)	17% (9/52)	

TILs*	**Present (moderate and marked)**	74% (31/42) [*CS*] 50% (8/16)^31^	24% (338/1406)	58% (155/268)	33% (4/12)
	
	**Marked**	17% (7/42) [*CS*]	4% (34/889)	29% (63/218)	17% (2/12)

***APC ***	**Mutations (^†^MCR, ^‡^also outside the MCR)**	14% (5/36) [*CS*]† 43% (6/14)^11‡^ 83% (5/6)^31†^	63% (459/724)^† ^* 52% (281/539)^‡^	5% (1/21)^†^ 41% (56/136)^‡^	33%(6/18)^‡^

***KRAS ***	**Mutations codon 12/13**	64% (23/36) [*CS*] 64% (9/14)^11^	29% (1090/3710)	20% (56/280)	34% (91/267)

**Beta-catenin (*CTNNB1*)**	**Nuclear staining**	11% (4/35) [*CS*] 71% (12/17)^11^	77% (138/179)	13% (4/31)	59% (40/68)
	
	**Mutations**	0% (0/16)^5^	5% (30/610)	7% (2/27)	20% (11/56)

***P53***	**Nuclear staining** **>25%**	34% (12/35) [*CS*] 53% (8/15)^11^	57% (668/1167)	15% (20/130)	72% (23/32)
	
	**Mutations**	60% (9/15) [*CS*] 21% (3/14)^11^	43% (1808/4299)	22% (21/95)	22% (2/9)

***SMAD4***	**Mutations**	26% (5/19) [*CS*] 0% (0/14)^11^	22% (17/77)		18% (2/11)

**MSI**	**High**	0% (0/33) [*CS*] 0% (0/17)^11^ 18% (2/11)^31^ 33% (1/3)^43^	12% (227/1834)		90% (88/98)

CS = current study, MAP = MUTYH-associated polyposis, MSS= microsatellite stable, MSI = microsatellite instability, TIL = Tumour Infiltrating Lymphocytes, MCR = mutation cluster region. *Relative large proportion of missense mutations (29%) in study by Luchtenborg et al as compared to other studies, explaining the high overall percentage of *APC *mutations in the MCR only group. See Additional file 1 online for more details and article references.

**Table 3:** Concise overview of data in Table 2

	MAP	Sporadic CRC	Sporadic MSI-high	Lynch (based on MMR mutations)
**Average age (years)**	49	68	67–75	47

**MAC stage C or D**	+	++	++	+

**Proximal location**	++	+	+++	++

**(Meta) synchronous CRC**	+	0	ND	+

**Poor Differentiation**	+	0	+	+

**Mucinous (>50%)**	+ *	+ *	+	+

**Crohn's like infiltrate (conspicuous)**	+	+	++	+

**Necrosis**	+	++	+	ND

TILs* present	++	+	++	+

**TILs marked**	+	0	+	+

***APC*-MCR mutations**	+	++	+	+

***KRAS* mutations (codon 12/13)**	++	+	+	+

**Beta-catenin (nuclear staining)**	+	+++	+	++

**Beta-catenin (*CTNNB1) *mutations**	0	0	0	+

**P53 (nuclear staining >25%)**	+	++	+	+++

***P53* mutations**	++	++	+	+

***SMAD4 *mutations**	+	+	0	+

**MSI-high**	0	+	+++	+++

0 = 0–10%, + = 11–40%, ++ = 41–70%, +++ = >70% ND = no data

* mucinous rate in MAP CRCs in this study was two times more than in sporadic CRCs: 23% and 12% respectively (see also table 2).

No significant geno-phenotype correlations for any of the main histopathological parameters could be found.

### Somatic mutation analysis and immunohistochemical staining

*APC *mutation analysis (Table 4) of the mutation cluster region showed somatic mutations in 5/36 (14%) carcinomas; four were *MUTYH *associated transversions (G>T's); two were C>T transitions, one of them occurring together with a G>A transition (patient 6). *KRAS2 *mutations were found in 23/36 (64%) of tumours, 22 were c.34G>T transversions. An increased nuclear and reduced membranous beta-catenin staining was found in 11% (4/35). In 57% (20/35) of MAP CRCs, p53 staining indicative of a functional p53 status (>0<25% nuclear staining) was found. Nuclear staining indicative of p53 dysfunction was found in 34% (12/35) (Figure 1E). In 9 out of 16 carcinomas (56%) that could be analyzed, ten *p53 *mutations were found. One carcinoma had two mutations (patient 7, Table 4). Three mutations were G>T transversions. Except in one case (patient 7, Table 4), staining was in concordance with the combined results of the p53 staining and LOH of chromosome 17p results published previously by Middeldorp et al (Table 4).[13] When staining was indicative of a dysfunctional p53 status, a mutation as well as LOH was found (patients 2, 23, 24, and 41). In cases were a mutation is present but no LOH was identified for 17p, staining was indicative of a still intact, functional p53 (patient 5, 8, and 16). Only one case (patient 22) had a nonsense mutation in *p53*, explaining the absence of nuclear staining. All other *p53 *mutations are (probable pathogenic) amino acid substitutions and all except one have been published previously [29](Table 4). *SMAD4 *mutations were present in 26% of MAP carcinomas tested (5/19, Table 4). Two tumours had G>T tranversions.

**Table 4:** Results of somatic mutation analysis and IHC analysis

*Tumour * *nr*	*APC (MCR)* mutation*	*KRAS * *mutation*	*p53 * *IHC†*	p53 mutation*	*17P* *LOH*^@^	*Beta-catenin * *IHC††*	SMAD4 mutation*	*MSI*^*c*^	*MLH1-PMS2* IHC**
1	no	c.34G>T	++	No	yes	0	c.227G>GT, p.R76RI	S	+

2	no	c.34G>T	+++	c.758C>CT, p.T253TI*	yes	0	no	S	+

3	no	no	+++		yes	0		S	+

4	no	c.34G>T	+		no	0	c.1058A>AG, p.Y353YC* c.1096C>CT, p.Q366QX	S	+

5	no	c.34G>T	+	c.593A>AT, p.E198EV	no	0	c.161T>TC, p.L54LS c.740G>GA, p.G247GE c.1597C>CT, p.L533LF	S	+

7	c.3949G>GT p. E1317EX* c.4339C>CT p. Q1447QX*	no	+	c.565G>GA, p.A189AT* c.599A>AG, p.N200NS*	yes	0/+	no	S	+

8	no	c.34G>T	+	c.446C>CT, p.S149SF*	no	0	no	S	+

11	no	no	0			0		S	+

12	no	no	+			0		S	+

13	no	c.34G>T	0			+/++		L~	heterogenous

14	no	no		No	no		no	S	+

16	no	c.34G>T	+	c.446C>CT, p.S149SF*	no	+	c.115G>GA, p.A39AT c.74G>GA, p.C25CY	S	+

17	no	c.34G>T	+++		no	0	c.1609G>GT, p.D537DY*		+

18	no	no	+			0		S	

20	no	c.34G>T	+			0/+		S	+

21	c.4222G>GT p.E1408EX*	no	+			0		S	+

22	c.4222G>GT p.E1408EX*	no	+	c.13791G>GT, p.E271EX*	yes	0/+	no	S	+

23	no	c.34G>T	++	c.596G>GT, p.G199GV*	yes	0	no	S	+

24	no	c.34G>T	++	c.820G>GT, p.V274VF*	yes	0		S	+

28	no	c.34G>T	+	No		0	no	S	+

29	no	c.34G>T	0		yes	0/+	no	S	+

30	no	c.34G>T	+			0/+		S	+

31	c.4085C>CT p.S1362SF	no	+			++		S	+

32	no	no	+++		no	+	no	S	

33	no	c.34G>T	++			0/+		S	

34	no	no	+++	No	yes	0	no	S	+

35	no	c.34G>T	+	No	no	0		S	+

36	c.4381G>GT p.E1461EX	c.34G>T	+		no	0/+	no	S	+

37	no	c.34G>T	+		yes	0		S	

38	no	no	+		no	0/+		S	+

39	no	c.34G>T	+			0		S	+

40	no	c.34G>T	+			0		S	+

41	no	no	++	c.13794G>GA, p.V272VM*	yes	0	no	S	+

42	no	c.34G>A	++			0	no	S	+

43	no	c.34G>T	+	no	yes	0	no	S	+

44	no	c.34G>T	++			0/+		S	+

Blank cells: not done/not ascertainable, † 0 = none, + = >0<25%, ++ = 25–75%, +++>75%, *previously reported mutations, see http://www.sanger.ac.uk/genetics/CGP/cosmic/ (*SMAD4*) and http://p53.free.fr/index.html (*P53*), ^@^LOH, as reported by Middeldorp et al (mainly copy neutral LOH and not physical loss),^8 ^†† 0= category 1(membranous staining), 0/+ = 2A (membranous and some nuclear staining), + = 2B (membranous & increased nuclear staining), ++ = 3 (strong nuclear & less or no membranous staining), ~2/9 markers unstable.

### Infiltrate analysis

**Figure 2:**
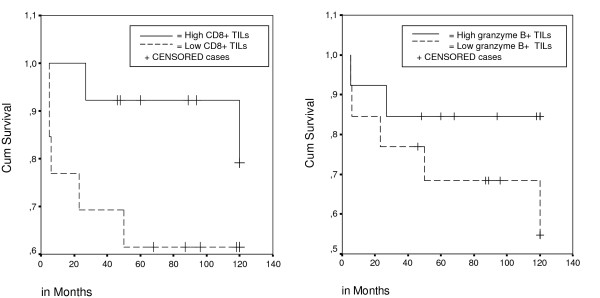
**Survival in 26 MAP patients, according to CD8^+ ^status and granzyme B^+ ^status**. high versus low, median, number of TILs as cut off point. Log rank; *P *= 0.15 (CD8^+^) and Log rank; *P *= 0.2 (granzyme B^+^).

First, we scored the presence of TILs and Crohn's like infiltrate on standard H&E sections. Secondly, in order to establish an objective lymphocytic cell count, we also performed triple-fluorescent IHC staining for CD3^+^, CD8^+^, and CD57^+ ^TILs (Figure 1F). The median number of intra-epithelial T-helper (CD3^+^, CD8^-^), cytotoxic T lymphocyte (CTL, CD8^+^, CD57^-^) and natural killer cells (NK, CD8^+^/CD57^+^) found in 34 CRCs available for analyses were 20, 37, and 0 cells/mm^2 ^tumour, respectively. There was a significant correlation between the total number of immunofluorescently detected TILs and the amount of TILs (none, present, marked) assessed on H&E slides (Spearman's test, *P *= 0.002). Immunohistochemistry for granzyme B, which is expressed on activated CD8+ cytotoxic lymphocytes (CTLs) and involved in the induction of apoptosis of target cells, showed a median of 4 granzyme B^+ ^cells/mm^2^. The number of CD8^+ ^TILs showed a significant positive correlation with the number of granzyme B^+ ^cells (Spearman's test, *P *= 0.009). No significant differences in survival were seen between patients with high versus low levels of TILs, although in patients with a high level of CD8^+ ^TILs and granzyme B^+ ^cells, a tendency was observed towards a better prognosis (Figure 2A and 2B, *P *= 0.15 and *P *= 0.2, respectively, log rank test).

### Literature Review

Results are shown in Table 2 and 3; different study outcomes were aggregated in Table 2 and Table 3 shows concise conclusions of these data. Data on Lynch syndrome carcinomas only included proven mismatch repair gene mutation carriers. An extended version of Table 2 is available online, as a supplement (Additional file 1), showing outcomes of all the individual articles and including data for Lynch syndrome suspected carcinomas where no germline mutations in the mismatch repair genes were searched for or found.

## Discussion

Specific histological and molecular characterization of CRCs may have implications in the diagnosis, prognosis, and treatment strategies, as demonstrated for mismatch repair deficient MSI-high CRCs.[15,16,30] In order to explore similar implications for MAP, we analyzed clinical, histological and molecular pathologic characteristics in our series of MAP CRCs and compared our findings to the literature (Table 2 and 3). Age at diagnosis of CRC, when compared to sporadic cases, was relatively young in MAP patients and comparable to that in Lynch patients (49 and 47 years). MAP CRCs showed less metastases than sporadic CRCs, but more than Lynch carcinomas (Table 2 and 3). A prominent feature is the 69% proximal sub-localization of colon carcinomas in this cohort; others reported a proximal location in 29, 43 and 46% of MAP CRCs.[4,11,31] This is still more than that reported in sporadic carcinomas (23%) but less than MSI-high or Lynch carcinomas (75% and 67% respectively, Table 2 and 3). Furthermore, this study showed that proximal MAP tumours had a preferential location in the cecum or ascending colon, as opposed to the transverse colon, which was also reported by O'Shea at al.[31]

The relatively high number of observed (meta) synchronous carcinomas in MAP patients is in agreement with previous reports on MAP patients (Table 2). In contrast to sporadic carcinomas but in agreement with MSI-high carcinomas, MAP CRC's were demonstrated to be relatively often mucinous in this study (Table 2). O'Shea et al. found a poor (low) differentiation grade in all MAP carcinomas (16/16) but remarkably, none had a mucinous pattern (0/16).[31]

In MSI-high tumours the caretaker function of mismatch repair enzymes is disrupted resulting in somatic mutations that are accumulated throughout the cell genome. Such abundance of mutations can result in aberrant frameshift peptides that would be presented at the cell surface, through the antigen processing pathway, to cells of the immune system. This sequence of events could explain the presence of an accentuated intra-epithelial lymphocytic infiltrate in MSI-high tumours.[14,32,33] In MAP carcinomas the disruption of the caretaker function of the BER machinery, mediated by *MUTYH *mutations, leads to the accumulation of G>T somatic mutations, at least early in tumourigenesis, which might evoke similar specific anti-tumour immune responses. TILs were present in a majority of MAP CRCs (74%) in this study, although marked infiltration was only detected in about one fifth of MAP carcinomas. The median number of CD8^+ ^lymphocytes in MAP CRCs, scored with IHC, fell somewhere between that found in MSI-stable and MSI-high CRC's (indicated by absent MLH-1 expression) previously analyzed with the same procedure in our laboratory.[34] O'Shea et al. reported the same percentage of TILs present in MAP CRCs as in their controls (50%, 8/16).[31] Their controls, however, might not be representative of sporadic CRCs, as the number of TILs reported by others in sporadic CRCs is much less (24%, Table 2 and 3). In MSI-high tumours, an active immune response by the host (represented by a high number of TILs) was also associated with a better survival, especially when associated with granzyme B positivity.[33] The group of MAP patients with a high number of CD8^+ ^and granzyme B^+ ^TILs showed a better overall survival, although not statistically significant for either parameters.

The number of *APC *mutations in the mutation cluster region found in MAP carcinomas (14%) is notably lower than that reported in sporadic CRCs (63%, Table 2 and 3). One possible explanation for the relatively small number of APC mutations could be that in MAP CRCs more mutations lay outside the mutation cluster region. In sporadic CRCs, *APC *mutations in the MCR represent 50–77% of all *APC *mutations found. [35-37] In MAP tumours (CRCs and adenomas), a substantial proportion (~60%) of mutations were found outside the MCR.[10,11] Similarly, in MSI-high CRCs noticeably more APC mutations are found when larger regions of APC were analyzed (Table 2A). Another explanation might be that distal carcinomas, which are underrepresented in our cohort, have more APC mutations than proximal tumours as shown by Luchtenborg et al.[38] In addition, *APC*-MCR mutations were seen in 46% (13/28) of rectum carcinomas analyzed previously in our laboratory.[39]

In agreement with data derived on MSI-high carcinomas, strong beta-catenin nuclear staining was not frequent in MAP carcinomas in this study (13% and 11%, respectively). In sporadic CRCs this rate is much higher (77%, Table 2 and 3).

We found a high percentage of *KRAS2 *mutations in our MAP CRCs (64%), comparable to reports by Lipton et al. and Jones et al.[11,40] The vast majority (96%) were c.34G>T transversions (GGT>TGT). Intriguingly, no G>T transversions at the second nucleotide of this codon (leading to GGT> GTT) have been reported so far in MAP CRCs.

In contrast, *KRAS2 *mutations are found on average in 29% of sporadic CRCs and 22% of sporadic MSI-high carcinomas. Furthermore, the c.34G>T tranversion comprises just 8% of *KRAS2 *mutations in sporadic and none in MSI-high CRCs.[12] In Lynch carcinomas the percentage of *KRAS2 *mutations is around 34%, Table 2 and 3) and in these carcinomas other hotspot mutations are found, namely the c.35G>A and c.38G>A, compromising 81% of detected *KRA2S *mutations in these tumours. [41] Previously we have shown that *KRAS2 *hotspot analysis can be used to detect MAP tumours.[5] Since *KRAS2 *mutations have been found previously in aberrant crypt foci (ACF) as well,[42] the high prevalence of *KRAS2 *mutations might influence tumourigenesis in MAP.

Nuclear staining indicative of p53 dysfunction was found in 34% in this study, which is less than found in sporadic carcinomas (57%, Table 2A). We found *p53 *mutations in 60% of carcinomas analyzed; this finding, along with the presence of LOH, correlated with the IHC staining results (Table 4). Three mutations were typical *MUTYH *G>T transversions. Lipton et al. found nuclear staining in 53% of MAP carcinomas, but *p53 *mutations in only 21% (of which a minority were G>T transversions), suggesting an alternative mechanism of over-expression.[11] The employment of tissue arrays for the current study could imply an underestimation of cases with both p53 and beta-catenin accumulation in the nucleus. However, we reported that 3 punches representing one tumour in a TMA correctly recapitulated the observations made on analysis of the whole slides.[27].

*SMAD4 *somatic mutations in this series were present in 26% (5/19, Table 4). Lipton et al. did not find any *SMAD4 *mutations in MAP CRCs, but did find 18q LOH at the same frequency as in sporadic CRCs.[11] Recently we reported that the chromosome 18q LOH in MAP carcinomas mainly comprises copy neutral LOH and not physical loss, as observed in sporadic CRC.[13]

In the early stages of MAP tumourigenesis, a dominance of the BER defect can be concluded from the high frequency of G>T tranversions in *KRAS2 *and *APC*. In the later stages such G>T transversions seem less prominent, as seen in *SMAD4 *and *p53*. Mitotic recombination might be a driving force in MAP carcinogenesis, based on our conclusion that the LOH in MAP carcinomas mainly comprise copy neutral LOH. Two previous studies by Colebatch and Lefevre et al.[43,44] have suggested that MAP CRCs can also develop through a MSI pathway (by inactivation of *MLH1*) because of the finding of MSI-high phenotype in one out of three and one out of six MAP CRCs, respectively. O'Shea et al. found MMR deficiency in 2 out of 11 tumours (18%). However, on the basis of the results of this study (an absence of MSI-high in 35 analyzed carcinomas), we conclude that the MSI pathway is not an important pathway in the development of MUTYH associated tumours.

## Conclusion

MAP CRCs as a group show specific histological features that differentiate them from sporadic CRCs, and have similarities with sporadic MSI-high and Lynch syndrome colon cancers, such as a preferential proximal location, mucinous histotype, and increased presence of TILs. These TILs might suggest that defects in base excision repair, similar to mismatch repair deficiency, produce secondary aberrant proteins functioning as tumour-specific neoantigens that, in turn, induce anti-tumour immune responses. Further evidence for MAP can be assembled by the detection of c.34G>T transversion in *KRAS2 *that takes place in early tumour development. *KRAS2 *analysis can be implemented as a pre-screening test that helps selecting CRC patients eligibly for germline *MUTYH *mutation testing. In practice, above features should direct the pathologist towards a MAP aetiology of CRC as an alternative for a mismatch repair deficient cause, especially when diagnosed at a young age and in combination with polyps and/or a recessive inheritance pattern.

## Competing interests

The authors declare that they have no competing interests.

## Authors' contributions

MN, NFCCM, MvP, AM, RvE and CMJT carried out the molecular genetic studies. MN, NFCCM, MvP, AM and RvE carried out the sequence analyses. MN, MvP, RvE, CMJT, AM, HFAV, FJH and HM carried out the acquisition, analysis and interpretation of data. MN and NFCCM performed the statistical analysis. MN, NFCCM, HFAV, FJH and HM drafted the manuscript. ESJ carried out the immunoassays. MN, NFCCM, ESJ, TvW, FJH and HM participated in the design of the study. NFCCM, ESJ, TvW, HFAV, FJH and HM revised the manuscript critically for important intellectual content. HM participated in its design and coordination. All authors read and approved the final manuscript.

## Pre-publication history

The pre-publication history for this paper can be accessed here:

http://www.biomedcentral.com/1471-2407/9/184/prepub

## Supplementary Material

Additional file 1 **Histological and molecular features in carcinomas, extended version of **table 2. Blank cells: not done/not ascertainable, †0 = none, + = >0<25%, ++ = 25–75%, +++>75%, *previously reported mutations, see http://www.sanger.ac.uk/genetics/CGP/cosmic/ (SAMD4) and http://p53.free.fr/index.html (P53), ^@^LOH, as reported by Middeldorp et al (mainly copy neutral LOH and not physical loss),^8 ^††0 = category 1(membranous staining), 0/+ = 2A (membranous and some nuclear staining), + = 2B (membranous & increased nuclear staining), ++ = 3 (strong nuclear & less or no membranous staining), **+ = nuclear staining, heterogenous= absent nuclear staining in part of tumour tissue, 0= no nuclear staining ±/± = weak nuclear staining, ~ 2/9 markers unstable. Click here for file
