# Supplementary material for: Colorectal carcinomas in MUTYH-associated polyposis display histopathological similarities to microsatellite unstable carcinomas
Source: BMC Cancer. 2009 Jun 15;9:184. doi: 10.1186/1471-2407-9-184 (PMC2706846; doi:10.1186/1471-2407-9-184)
Supplement: Additional file 1 — Histological and molecular features in carcinomas, extended version of table 2. Blank cells: not done/not ascertainable, †0 = none, + = >0<25%, ++ = 25–75%, +++>75%, *previously reported mutations, see http://www.sanger.ac.uk/genetics/CGP/cosmic/ (SAMD4) and http://p53.free.fr/index.html (P53), @LOH, as reported by Middeldorp et al (mainly copy neutral LOH and not physical loss),8 ††0 = category 1(membranous staining), 0/+ = 2A (membranous and some nuclear staining), + = 2B (membranous & increased nuclear staining), ++ = 3 (strong nuclear & less or no membranous staining), **+ = nuclear staining, heterogenous= absent nuclear staining in part of tumour tissue, 0= no nuclear staining ±/± = weak nuclear staining, ~ 2/9 markers unstable. [file 1471-2407-9-184-S1.doc]

Additional file 1. Histological and molecular features in carcinomas, extended version

| **Characteristics**  **Colon carcinomas** |  | **MAP** | **Sporadic** | **Sporadic MSI-high** | **Lynch (based on family history*,**  **or on MMR mutations^+^)** |
| --- | --- | --- | --- | --- | --- |
| **Age at CRC** |  | 49 years^[CS]^  55 years^[1]^ | 68 years^39^ | 67 years^10^  75 years^15^ | 47 years^15^ |
| **TNM stage** | **III or IV** | 34% (51/148)^[59, includes CS]^  55% (10/18)^[1]^ | 42% (1781/4193)^39^ | 43% (19/44)^9^ | 32% (12/38)^5+^  20% (13/63)^16^* |
| **Proximal location** |  | 69% (35/51)^[CS]^  29% (7/24)^1^  43% (16/37)^2^  46% (6/13)^3^ | 19% (976/5138)^4^  30% (239/810)^5^  34% (218/645)^6^  18% (34/194)^7^  29% (233//804)^8^  40% (68/172)^9^  32% (119/366)^10^ | 79% (26/33)^11^  83% (15/18)^12^  50% (6/12)^13^  82% (10/12)^14^  32% (6/19)^7^  84% (43/51)^15^  74% (120/162)^8^  91% (40/44)^9^  72% (42/60)^10^ | 77% (44/57)^16^*  54% (30/56)^4^*  58% (22/38)^5+^  74% (45/61)^17+^  68% (62/91)^15^* ^or +^  58% (7/12)^10+^ |
| **CRC (Meta) synchronous** |  | 23% (10/44)^[CS]^  33% (6/18)^1^  26% (8/29)^2^ | 2% (14/832)^5^ |  | 18% (7/38)^5+^ |
| **Poor differentiation** |  | 38% (16/42)^[CS]^  22% (5/23)^1^  100% (16/16)^3^ | 5% (5/106)^14^  7% (381/5138)^4^  16% (126/776)^5^  17% (96/572)^6^  7% (14/194)^7^  18% (143/804)^8^ | 26% (5/19)^7^  25% (17/69)^10^  33% (11/33)^11^  38% (35/92)^18^  57% (27/47)^15^  38% (62/162)^8^  60% (28/47)^19^ | 13% (7/56)^4^*  31% (16/51)^20^* ^or +^  27% (10/37)^5+^  34% (28/81)^15^* ^or +^  44% (28/64)^17+^ |
| **Mucinous** | **(>50%)** | 21% (9/42)^[CS]^  13% (3/23)^1^  0% (0/16)^3^ | 6% (30/474)^21^  6% (18/300)^22^  12% (49/413)^10^  15% (121/798)^5^  12% (23/194)^7^  15% (22/147)^4^  17% (30/172)^9^ | 15% (14/92)^18^  23% (12/52)^21^  24% (16/67)^10^  31% (10/32)^11^  38% (18/47)^19^  36% (16/44)^9^  43% (18/42)^15^ | 14% (7/51)^20^* ^or +^  35% (13/37)^5+^  23% (13/56)^4^*  22% (16/72)^15^* ^or +^  33% (4/12)^10+^  36% (23/64)^17+^ |
| **Crohn’s like**  **infiltrate** | **Conspicuous** | 33% (13/40)^[CS]^  31% (5/16)^3^ | 10% (18/172)^9^  22% (180/804)^8^  27% (113/413)^10^  28% (28/100)^23^  36% (48/133)^18^  44% (202/4550)^21^ | 33% (11/33)^11^  48% (33/69)^10^  49% (35/71)^18^  48% (22/45)^15^  56% (90/162)^8^  50% (22/44)^9^  68% (47/69)^10^  69% (36/52)^21^ | 42% (5/12)^10+^  50% (32/64)^17+^  61% (48/79)^15^* ^or +^  71% (36/51)^20^* ^or +^ |
| **Necrosis** |  | 40% (16/40)^[CS]^ | 77% (356/465)^21^ | 17% (9/52)^21^ |  |
| **TIL** | **Present (moderate + marked)** | 74% (31/42)^[CS]^  50% (8/16)^3^ | 15% (41/275)^11^  24% (96/405)^10^  23% (109/468)^21^  35% (97/276)^24^ | 62% (42/68)^10^  56% (24/45)^15^  66% (22/33)^11^  61% (11/18)^20^  90% (47/52)^21^ | 33% (4/12)^10+^  73% (58/79)^15^* ^or +^  80% (41/51)^20^* ^or +^ |
|  | **Marked** | 17% (7/42)^[CS]^ | 3% (6/231)^18^  4% (11/271)^25^  4% (18/405)^10^ | 21% (19/92)^18^  28% (19/68)^10^  43% (25/58)^25^ | 17% (2/12)^10+^ |
| ***APC*** | **Mutations (^†^MCR, ^‡^also outside the MCR)** | 14% (5/36)^[CS]†^  43% (6/14)^1‡^ | 66% (439/665)*,only trunc.37% (248/665)^26†^  34%,(20/59)^27†^  35% (14/40)^28‡^  42% (77/184),only trunc. 36% (63/184)^29‡^  45% (34/76)^30‡^  50% (29/58)^31 ‡^  70% (127/181)^32‡^ | 5% (1/21)^27†^  11% (1/9)^33‡^  23% (5/22)^34‡^  34% (10/29)^32^)^‡^  46% (11/24)^28‡^  56% (29/52)^35‡^ | 27% (4/15)^27^* ^or +†^  25% (3/12)^36 ‡+^  50% (3/6)^37‡+^  57% (8/14)^38^*^‡^ |
| ***KRAS2*** | **Mutations codon 12/13** | 64% (23/36)^[CS]^  64% (9/14)^1^ | 27% (723/2721)^39^  36% (235/656)^6^  42% (82/194)^7^  35% (35/101)^30^  39% (15/38)^28^ | 0% (0/23)^15^  8% (2/24)^28^  18% (4/22)^34^  22% (2/9)^33^  22% (37/166)^58^  24% (4/17)^40^  37% (7/19)^7^ | 0% (0/9)^30+^  10% (2/20)^15^* ^or +^  17% (4/23)^41^*  28% (11/39)^42+^  61% (11/18)^38^*  40% (63/158)^58+^ |
| **Beta-catenin (*CTNNB1*)** | **Nuclear staining/**  **overexpression** | 11% (4/35)^[CS]^  71% (12/17)^1^ | 39% (17/44)^43^  73% (38/52)^44^  100% (83/83)^45^ | 13% (4/31)^15^ | 34% (29/86)^15^* ^or +^  46% (17/37)^42+^  74% (23/31)^46+^ |
|  | **Mutations** | 0% (0/16)^1^ | 1% (5/464)^6^  1% (1/72)^47^  15% (6/40)^48^  53% (18/34)^30^+ | 0% (0/22)^34^  40% (2/5)^30^ | 18% (8/44)^42+^  25% (3/12)^36+^ |
| ***P53*** | **Nuclear staining**  **>25%** | 34% (12/35)^[CS]^  53% (8/15)^1^ | 47% (186/389)^49^  53% (86/161)^34^  54% (93/172)^9^  60% (22/37)^50^  64% (113/176)^25^  70% (63/90)^51^  74% (105/142)^52^ | 5% (1/22)^34^  8% (3/34)^49^  20% (6/30)^15^  23% (10/44)^9^  27% (12/44)^25^ | 8% (6/75)^15^ * ^or +^  23% (5/22)^41^*  64% (7/11)^38^*^‡^  72% (23/32)^42+^ |
|  | **Mutations** | 60% (9/15)^[CS]^  21% (3/14)^1^ | 36% (13/36)^28^  42% (1,449/3,474)^53^  46% (90/194)^7^  45% (88/194)^40^  51% (73 /142)^52^  59% (95/160)^34^ | 5% (1/22)^34^  14% (2/14)^40^  27% (6/22)^28^  31% (4/13)^33^  32% (6/19)^7^  40% (2/5)^30^ | 17% (4/22)^41^*  22% (2/9)^30+^ |
| ***SMAD4*** | **Mutations** | 26% (5/19)^[CS]^  0% (0/14)^1^ | 22% (17/77)^36^ |  | 18% (2/11)^36+^ |
| **MSI** | **High** | 0% (0/35)^[CS]^  18% (2/11)^3^  0% (0/17)^1^ | 8% (28/336)^54^  9% (19/213)^7^  11% (12/106)^14^  10% (10/100)^42^  12% (63/509)^55^  17% (95/570)^56^ |  | 67% (20/30)^5+^  100% (43/43)^42+^  100% (25/25)^57+^ |

CS= current study, MAP=MUTYH-associated polyposis, FAP= familial adenomatous polyposis, MSS= microsatellite stable, MSI= Microsatellite instability, TILs= Tumour Infiltrating Lymphocytes, MCR= mutation cluster region.

* This study by Luchtenborg et al contained a relatively large proportion (29%) of missense mutations (of which the pathogenicity remains to be elucidated) as compared to other studies. Silence mutations were excluded.

**References**

1. Lipton L, Halford SE, Johnson V, et al. **Carcinogenesis in MYH-associated polyposis follows a distinct genetic pathway.** Cancer Res 2003;63:7595-7599.

2. Aretz S, Uhlhaas S, Goergens, et al.. **MUTYH-associated polyposis: 70 of 71 patients with biallelic mutations present with an attenuated or atypical phenotype**. Int J Cancer 2006;119:807-814.

3. O'Shea AM, Cleary SP, Croitoru MA, et al. **Pathological features of colorectal carcinomas in MYH-associated polyposis**. Histopathology 2008.

4. You JF, Hsieh LL, Changchien CR, et al. **Inverse effects of mucin on survival of matched hereditary nonpolyposis colorectal cancer and sporadic colorectal cancer patients.** Clin Cancer Res 2006;12:4244-4250.

5. Barnetson RA, Tenesa A, Farrington SM, et al. **Identification and survival of carriers of mutations in DNA mismatch-repair genes in colon cancer**. N Engl J Med 2006;354:2751-2763.

6. Luchtenborg M, Weijenberg MP, Wark PA, et al. **Mutations in APC, CTNNB1 and K-ras genes and expression of hMLH1 in sporadic colorectal carcinomas from the Netherlands Cohort Study.** BMC Cancer 2005;5:160.:160.

7. Chang SC, Lin JK, Yang SH, et al. **Relationship between genetic alterations and prognosis in sporadic colorectal cancer.** Int J Cancer 2006;118:1721-1727.

8. Jenkins MA, Hayashi S, O'Shea AM, et al. **Pathology features in Bethesda guidelines predict colorectal cancer microsatellite instability: a population-based study**. Gastroenterology 2007;133:48-56.

9. Gafa R, Maestri I, Matteuzzi M, et al. **Sporadic colorectal adenocarcinomas with high-frequency microsatellite instability.** Cancer 2000;89:2025-2037.

10. Yearsley M, Hampel H, Lehman A, et al. **Histologic features distinguish microsatellite-high from microsatellite-low and microsatellite-stable colorectal carcinomas, but do not differentiate germline mutations from methylation of the MLH1 promoter.** Hum Pathol 2006;37:831-838.

11. Ward R, Meagher A, Tomlinson I, et al. **Microsatellite instability and the clinicopathological features of sporadic colorectal cancer.** Gut 2001;48:821-829.

12. Dolcetti R, Viel A, Doglioni C, et al. **High prevalence of activated intraepithelial cytotoxic T lymphocytes and increased neoplastic cell apoptosis in colorectal carcinomas with microsatellite instability.** Am J Pathol 1999;154:1805-1813.

13. Kets CM, Hoogerbrugge N, Bodmer D, et al. **Unfavorable pathological characteristics in familial colorectal cancer with low-level microsatellite instability.** Mod Pathol 2006;19:1624-1630.

14. Takemoto N, Konishi F, Yamashita K, et al. **The correlation of microsatellite instability and tumor-infiltrating lymphocytes in hereditary non-polyposis colorectal cancer (HNPCC) and sporadic colorectal cancers: the significance of different types of lymphocyte infiltration.** Jpn J Clin Oncol 2004;34:90-98.

15. Young J, Simms LA, Biden KG, et al. **Features of colorectal cancers with high-level microsatellite instability occurring in familial and sporadic settings: parallel pathways of tumorigenesis.** Am J Pathol 2001;159:2107-2116.

16. Rodriguez-Bigas MA, Vasen HF, Pekka-Mecklin J, et al. **Rectal cancer risk in hereditary nonpolyposis colorectal cancer after abdominal colectomy.** International Collaborative Group on HNPCC. Ann Surg 1997;225:202-207.

17. Shashidharan M, Smyrk T, Lin KM, et al. **Histologic comparison of hereditary nonpolyposis colorectal cancer associated with MSH2 and MLH1 and colorectal cancer from the general population.** Dis Colon Rectum 1999;42:722-726.

18. Alexander J, Watanabe T, Wu TT, Rashid A, Li S, Hamilton SR.. **Histopathological identification of colon cancer with microsatellite instability.** Am J Pathol 2001;158:527-535.

19. Guidoboni M, Gafa R, Viel A, et al. **Microsatellite instability and high content of activated cytotoxic lymphocytes identify colon cancer patients with a favorable prognosis.** Am J Pathol 2001;159:297-304.

20. Jass JR, Walsh MD, Barker M, et al. **Distinction between familial and sporadic forms of colorectal cancer showing DNA microsatellite instability.** Eur J Cancer 2002;38:858-866.

21. Greenson JK, Bonner JD, Ben Yzhak O, et al. **Phenotype of microsatellite unstable colorectal carcinomas: Well-differentiated and focally mucinous tumors and the absence of dirty necrosis correlate with microsatellite instability.** Am J Surg Pathol 2003;27:563-570.

22. Nozoe T, Anai H, Nasu S, Sugimachi K. **Clinicopathological characteristics of mucinous carcinoma of the colon and rectum.** J Surg Oncol 2000;75:103-107.

23. Graham DM, Appelman HD. **Crohn's-like lymphoid reaction and colorectal carcinoma: a potential histologic prognosticator.** Mod Pathol 1990;3:332-335.

24. Ropponen KM, Eskelinen MJ, Lipponen PK et al. **Prognostic value of tumour-infiltrating lymphocytes (TILs) in colorectal cancer.** J Pathol 1997;182:318-324.

25. Sinicrope FA, Rego RL, Foster N, et al. **Microsatellite instability accounts for tumor site-related differences in clinicopathologic variables and prognosis in human colon cancers.** Am J Gastroenterol 2006;101:2818-2825.

26. Luchtenborg M, Weijenberg MP, Roemen GM, et al. **APC mutations in sporadic colorectal carcinomas from The Netherlands Cohort Study.** Carcinogenesis 2004;25:1219-1226.

27. Jass JR, Barker M, Fraser L, et al. **APC mutation and tumour budding in colorectal cancer.** J Clin Pathol 2003;56:69-73.

28. Shitoh K, Konishi F, Miyaki M, et al. **Pathogenesis of non-familial colorectal carcinomas with high microsatellite instability.** J Clin Pathol 2000;53:841-845.

29. Diergaarde B, van Geloof WL, van Muijen GN, et al. **Dietary factors and the occurrence of truncating APC mutations in sporadic colon carcinomas: a Dutch population-based study.** Carcinogenesis 2003;24:283-290.

30. Konishi M, Kikuchi-Yanoshita R, Tanaka K, et al. **Molecular nature of colon tumors in hereditary nonpolyposis colon cancer, familial polyposis, and sporadic colon cancer.** Gastroenterology 1996;111:307-317.

31. De Filippo C, Luceri C, Caderni G, et al. **Mutations of the APC gene in human sporadic colorectal cancers.** Scand J Gastroenterol 2002;37:1048-1053.

32. Lovig T, Meling GI, Diep CB, et al. **APC and CTNNB1 mutations in a large series of sporadic colorectal carcinomas stratified by the microsatellite instability status.** Scand J Gastroenterol 2002;37:1184-1193.

33. Olschwang S, Hamelin R, Laurent-Puig P, et al. **Alternative genetic pathways in colorectal carcinogenesis.** Proc Natl Acad Sci U S A 1997;94:12122-12127.

34. Salahshor S, Kressner U, Pahlman L, et al. **Colorectal cancer with and without microsatellite instability involves different genes.** Genes Chromosomes Cancer 1999;26:247-252.

35. Huang J, Papadopoulos N, McKinley AJ, et al. **APC mutations in colorectal tumors with mismatch repair deficiency.** Proc Natl Acad Sci U S A 1996;93:9049-9054.

36. Miyaki M, Iijima T, Konishi M, et al. **Higher frequency of Smad4 gene mutation in human colorectal cancer with distant metastasis.** Oncogene 1999;18:3098-3103.

37. Homfray TF, Cottrell SE, Ilyas M, et al. **Defects in mismatch repair occur after APC mutations in the pathogenesis of sporadic colorectal tumours.** Hum Mutat 1998;11:114-120.

38. Aaltonen LA, Peltomaki P, Leach FS, et al. **Clues to the pathogenesis of familial colorectal cancer.** Science 1993;260:812-816.

39. Andreyev HJ, Norman AR, Cunningham D, et al. **Kirsten ras mutations in patients with colorectal cancer: the 'RASCAL II' study**. Br J Cancer 2001;85:692-696.

40. Breivik J, Lothe RA, Meling GI, et al. **Different genetic pathways to proximal and distal colorectal cancer influenced by sex-related factors.** Int J Cancer 1997;74:664-669.

41. Losi L, Ponz DL, Jiricny J, et al. **K-ras and p53 mutations in hereditary non-polyposis colorectal cancers.** Int J Cancer 1997;74:94-96.

42. Johnson V, Lipton LR, Cummings C, et al. **Analysis of somatic molecular changes, clinicopathological features, family history, and germline mutations in colorectal cancer families: evidence for efficient diagnosis of HNPCC and for the existence of distinct groups of non-HNPCC families.** J Med Genet 2005;42:756-762.

43. Kobayashi M, Honma T, Matsuda Y, et al. **Nuclear translocation of beta-catenin in colorectal cancer.** Br J Cancer 2000;82:1689-1693.

44. Hao X, Frayling IM, Willcocks TC, et al. **Beta-catenin expression and allelic loss at APC in sporadic colorectal carcinogenesis.** Virchows Arch 2002;440:362-366.

45. Iwamoto M, Ahnen DJ, Franklin WA, et al. **Expression of beta-catenin and full-length APC protein in normal and neoplastic colonic tissues.** Carcinogenesis 2000;21:1935-1940.

46. Kariola R, Abdel-Rahman WM, Ollikainen M, et al. **APC and beta-catenin protein expression patterns in HNPCC-related endometrial and colorectal cancers**. Fam Cancer 2005;4:187-190.

47. Samowitz WS, Powers MD, Spirio LN, et al. **Beta-catenin mutations are more frequent in small colorectal adenomas than in larger adenomas and invasive carcinomas**. Cancer Res 1999;59:1442-1444.

48. Sparks AB, Morin PJ, Vogelstein B, et al. **Mutational analysis of the APC/beta-catenin/Tcf pathway in colorectal cancer.** Cancer Res 1998;58:1130-1134.

49. Elsaleh H, Powell B, McCaul K, et al. **P53 alteration and microsatellite instability have predictive value for survival benefit from chemotherapy in stage III colorectal carcinoma.** Clin Cancer Res 2001;7:1343-1349.

50. Kim GP, Colangelo LH, Wieand HS, et al. **Prognostic and predictive roles of high-degree microsatellite instability in colon cancer: a National Cancer Institute-National Surgical Adjuvant Breast and Bowel Project Collaborative Study**. J Clin Oncol 2007;25:767-772.

51. Cunningham J, Lust JA, Schaid DJ, et al. **Expression of p53 and 17p allelic loss in colorectal carcinoma.** Cancer Res 1992;52:1974-1980.

52. Kaserer K, Schmaus J, Bethge U, et al. **Staining patterns of p53 immunohistochemistry and their biological significance in colorectal cancer.** J Pathol 2000;190:450-456.

53. Russo A, Bazan V, Iacopetta B, et al. **The TP53 colorectal cancer international collaborative study on the prognostic and predictive significance of p53 mutation: influence of tumor site, type of mutation, and adjuvant treatment**. J Clin Oncol 2005;%20;23:7518-7528.

54. Percesepe A, Borghi F, Menigatti M, et al. **Molecular screening for hereditary nonpolyposis colorectal cancer: a prospective, population-based study**. J Clin Oncol 2001;19:3944-3950.

55. Aaltonen LA, Salovaara R, Kristo P, et al. **Incidence of hereditary nonpolyposis colorectal cancer and the feasibility of molecular screening for the disease.** N Engl J Med 1998;338:1481-1487.

56. Ribic CM, Sargent DJ, Moore MJ, et al. **Tumor microsatellite-instability status as a predictor of benefit from fluorouracil-based adjuvant chemotherapy for colon cancer**. N Engl J Med 2003;349:247-257.

57. Smyrk TC, Watson P, Kaul K, et al. **Tumor-infiltrating lymphocytes are a marker for microsatellite instability in colorectal carcinoma**. Cancer 2001;91:2417-2422.

58. Oliveira C, Westra JL**, Arango D,** et al. **Distinct patterns of KRAS mutations in colorectal carcinomas according to germline mismatch repair defects and hMLH1 methylation status**. Human Molecular Genetics 2004 13(19):2303-2311.

59. Nielsen M, Joerink - van de Beld M.C., Jones N.. et al. **Analysis of MUTYH Genotypes and Colorectal Phenotypes in Patients With MUTYH-Associated Polyposis.** Gastroenterology 2009; 136(2): 471-476
